# The inverse starving test is not a suitable provocation test for Gilbert's syndrome

**DOI:** 10.1186/1756-0500-1-35

**Published:** 2008-06-24

**Authors:** Niels Teich, Inken Lehmann, Jonas Rosendahl, Michael Tröltzsch, Joachim Mössner, Ingolf Schiefke

**Affiliations:** 1 Internistische Gemeinschaftspraxis für Verdauungs- und Stoffwechselerkrankungen, Leipzig, Germany; 2 Department of Gastroenterology, Medizinische Klinik und Poliklinik II, Universitätsklinikum Leipzig, Leipzig, Germany; 3 Praxisgemeinschaft für Innere Medizin und Radiologie, Markkleeberg, Germany

## Abstract

**Background:**

**Introduction:**

The aim of this study was to evaluate a simple diagnostic test for Gilbert's syndrome (GS), which avoids hospitalization and exposure to toxic test substrates. GS is the most frequent cause of isolated unconjugated hyperbilirubinemia. The nicotinic acid test and the starving test are established approaches to diagnose GS. However, these tests cause considerable side effects or require hospital admission. In single GS patients, we observed rapid serum bilirubin normalization after a standard European lunch (the "inverse starving test").

**Findings:**

At two consecutive days, 18 profoundly characterized GS patients (7 females, 11 males, median age 34.5 years, range 21–58 years) were investigated with the nicotinic acid test and the inverse starving test. Unconjugated serum bilirubin (UCB) levels were measured before and hourly up to four hours after lunch (median 645 kcal), and after the ingestion of 170 milligrams nicotinic acid, respectively. Patients who consulted their physicians with jaundice were significantly more likely to undergo invasive diagnostic procedures than patients with an incidental finding of elevated UCB, despite UCB levels were indifferent in both groups. Two hours after nicotinic acid ingestion, relative UCB exceeded 1.7 fold the fasting levels (median, range 0.9–2.4 fold, sensitivity 83%). In the inverse starving test, UCB remained almost unchanged three hours after lunch (median 1.0; range: 0.8–1.2 fold). Molecular analysis established the genotype of the TATAA box of the UGT1A1 gene; all patients carried an UGT1A1 promotor polymorphism.

**Conclusion:**

The inverse starving test is not an appropriate provocation test for patients with suspected GS. The 100% prevalence of the UGT1A1 polymorphism in our cohort underlines that the diagnosis of GS may be substantiated with this simple molecular test in patients with an uncertain diagnosis of GS.

## Findings

### Background

Conjugation with glucuronic acid is essential for efficient excretion of bilirubin. Bilirubin glucuronidation is mediated by a microsomal enzyme, the bilirubin uridine diphosphate glucuronosyltransferase (UGT1A1; EC 2.4.1.17) [1]. Gilbert's syndrome (syn. icterus juvenilis Meulengracht) is an inherited benign disorder characterized by unconjugated hyperbilirubinemia in the absence of structural liver disease or haemolysis [2]. The elevated unconjugated bilirubin (UCB) concentration is usually noted first in adolescence, either as an incidental finding or because of a slight yellow discoloration of the sclera. The serum bilirubin concentration characteristically fluctuates daily, occasionally falling within the normal range. During fasting, physical exercise, stress, intercurrent illness, or menstruation bilirubin exceeds the upper normal limit, sometimes by as much as four times. Gilbert's syndrome only occasionally causes critical symptoms, if treatment with cytostatic drugs as irinotecan is necessary. In these circumstances, live threatening complications as grade 4 neutropenia and diarrhoea may occur [3,4]. Furthermore, patients with a *UGT1A1 *promoter polymorphism are more likely to develop hyperbilirubinemia after treatment with the anti-retroviral protease inhibitor atazanavir [5].

GS prevalence ranges from 2% to 12% in different populations [6,7]. The inheritance of GS is either autosomal dominant with an incomplete penetrance or recessive. A promotor polymorphism of the TATAA box within the *UGT1A1 *gene is associated with GS in more than 50% of patients [8].

The clinical diagnosis of Gilbert's syndrome is made by exclusion of liver disease or overt haemolysis. Several provocation tests have been established to discriminate GS patients from patients with acute or chronic liver disease, haemolytic anemia and healthy volunteers.

The "**caloric restriction**" or "starving" test has been inaugurated by Augustine Gilbert itself [9]. It involves restricting the caloric intake to 400 kcal per 24 h for two days. This should double UCB from the baseline level and clearly distinct GS patients from healthy volunteers [10]. The discrimination from non-GS patients was insufficient, however, if patients with liver cirrhosis or acute hepatitis have been compared with GS patients [11,12]. Moreover, this test is time-consuming and requires hospital admission to control blood glucose levels and the exact caloric intake throughout the test period. Moreover, an evaluation for the presence of GS fails the "Appropriateness Evaluation Protocol" (AEP) criteria of admission to hospitals and is not reimbursed by the public health insurances in Germany.

The **nicotinic acid **test involves overnight fasting and intravenous (50 mg) or oral (170 – 300 mg) administration of nicotinic acid (NA) [13,14]. A significant increase of UCB is seen in GS patients. The most frequent adverse event of nicotinic acid is vasodilatation. It is frequently accompanied with tachycardia, headache, nausea and vomiting. Another adverse event is haemolysis, which is probably caused by a higher susceptibility of erythrocytes to splenic haemolysis, since the NA triggered UCB rise is abolished after splenectomy [15,16]. In a small series, indomethacin pre-treatment was able to reduce NA side effects without changing the hyperbilirubinemic effect of nicotinic acid [17]. All studies, which directly compared the starving test and the intravenous nicotinic acid test found a higher bilirubin increase in the latter [10,17].

**Rifampin **induces cytochrome P-450 isoenzymes and competes for the excretory pathways in liver cells. The "rifampin test" with 900 mg orally administered rifampin had the ability to distinguish GS patients from controls in one pilot study [18]. Actually, Hallal and colleagues confirmed the usefulness of the rifampin test in a study with 89 GS patients and pointed to the better diagnostic value of the relative rather than the absolute bilirubin increase after rifampin administration [19]. In both studies, conflicting findings regarding rifampin induced haemolysis were reported. Furthermore, both studies did not report on the frequency of adverse events after rifampin administration, such as heartburn, vomiting or even hepatitis. As well, severe complications such as disseminated intravascular coagulation or serious hypersensitivity reactions including the Stevens-Johnson Syndrome and toxic epidermal necrolysis may rarely occur after rifampin administration.

The aim of this study was to evaluate a simple diagnostic test for GS, which avoids hospitalization and exposure to toxic test substrates. Single case observations revealed a rapid decline of UCB in overnight fasting GS patients, if they have ingested a standard European lunch. This approach is inexpensive, non-toxic and practicable in an out-patients department. Deriving from these observations, we conducted this prospective study to evaluate the diagnostic accuracy of this "inverse starving test" in patients with GS. The test was pre-assigned to be clinical useful, if an UCB-decline of at least 50% could be achieved. So far, no gold standard for the diagnosis of GS has been established. As a reasonable control investigation, we used the nicotinic acid test, which should increase UCB by 50%.

## Methods

Between July and November 2006, we sent invitation letters to 59 patients with (suspected) GS. After comprehensive information, 20 patients matched the inclusion criteria and gave their written informed consent. Two patients were excluded from the study, as they matched a stop criterion: One 28-year-old male with remittent ulcerative colitis had a haptoglobin level <0,2 due to a so far unknown slight haemolysis (haemoglobin concentration = 7.6 mmol/L, normal range 8.2–10.7; lactate dehydrogenase 3.82 microkat/l, normal range 2.25–3.75). A 50-year-old female was excluded due to a liver nodule of 1 centimetre. Invasive diagnostics and a six months follow-up did not reveale malignancy so far. Furthermore, one 32-year-old female declined to continue the study after the sonographic finding of a fatty liver.

The diagnosis of GS was suspected at least three months before our investigation due to elevated unconjugated bilirubin levels, normal aspartate amino transferase (ASAT, GOT), alanine amino transferase (ALAT, GPT), gamma-glutamyl transferase (GGT), alkaline phosphatase, lactate dehydrogenase (LDH) and haptoglobine levels. Molecular analysis established the genotype of the TATAA box of the UGT1A1 gene by standard molecular techniques; all patients carried an UGT1A1 promotor polymorphism. The study was approved by the Ethics Committee of the University of Leipzig. This committee did not consent to expand our investigations to healthy controls. Exclusion and stop criteria are referred in the tables 1 and 2.

**Table 1:** Exclusion criteria

**Exclusion criteria**
nicotine incompatibility
history of haemolysis
history of myocardial infarction, coronary artery disease, cardiac arrhythmia or angina pectoris
Hyperthyroidism
pheochromocytoma
insulin dependent diabetes mellitus
arterial hypertension
kidney- or liver disease
patient's age under 18 or over 60 years
pregnancy and lactation
simultaneous participation in other clinical studies

**Table 2:** Stop criteria

**Stop criteria**
Solid liver lesion (other than typical cysts) or mechanic cholestasis in sonography
Elevated GOT, GPT, AP, GGT

The **inverse starving test **required an overnight fast. On arrival in the out-patients department, an initial blood sampling for conjugated and unconjugated bilirubin, ALAT, ASAT, GGT, lactate dehydrogenase, alkaline phosphatase and haptoglobine was done via an intravenous catheter. Within the next two hours, an abdominal ultrasound was carried out to exclude focal liver lesions and mechanic cholestasis. Two patients with abnormal findings in these investigations had to be excluded and were advised to undergo further diagnostics. 18 Patients without exclusion criteria got a free lunch of choice in the university restaurant after a second fasting blood sample was taken directly before lunch. After lunch, blood samples for bilirubin analysis were taken hourly up to 4 hours.

The **nicotinic acid test **was performed at the next day. After an overnight fast and fasting UCB analysis, the patients ingested 170 mg nicotinic acid with pure water. Further blood samples were taken in hourly intervals up to 4 hours. Between the repeated blood drawings via an intravenous catheter, the patients were advised to rest in the waiting room, and were only allowed to drink pure water.

### Statistical Analysis

All results were evaluated as median (interquartile range (IQR)) for continuous variables, and frequency counts for categorical variables. According to earlier publications a reduction greater than 1.5 relative UCB in starving test or an increase greater than 1.5 UCB in nicotinic acid test were assessed as clinical relevant. A sensitivity analysis was performed for all tests and time points.

## Results

18 GS patients completed both tests at two consecutive days. These included 11 males and 7 females with a median age of 34.5 years (range, 21–58), and a median body mass index of 22.1 kg/m^2 ^(range, 21.5–24). Two patients smoked 10 or 15 cigarettes per day, respectively, whereas the others were non-smokers. No patient regularly drinks alcohol, and none drunk alcohol within 24 hours before the tests and at the test days. None of the females used hormonal contraceptives. No patient had fever or felt unwell within one month before the tests.

The test patients had different initial symptoms. Yellow eyes (one with coincidental yellow skin) were noticed by three patients by themselves and in five cases by a relative. Fatigue was the first symptom in one case. In the remaining nine, elevated bilirubin levels were an incidental laboratory finding. The eight patients who consulted their doctors due to jaundice were significantly more likely to undergo liver biopsy (n = 4) or endoscopic retrograde cholangio-pancreaticography (ERCP, n = 1) than the nine patients with an incidental finding of elevated UCB (n = 0, p = 0.038, fisher exact test). Interestingly, fasting UCB was not different between these groups (p > 0.3, t-test).

Previously, all patients had ultrasound examinations; a computed tomography was done in three patients and magnet resonance tomography in two cases. Fasting UCB was not different between the patients who have undergone extensive radiological diagnostics and the patients with ultrasound only (p > 0.5). Bone marrow examination was not carried out in any case.

### The inverse starving test

**Figure 1:**
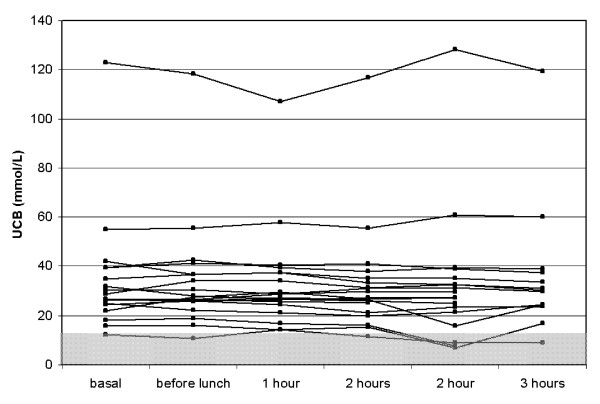
UCB course in the inverse starving test in the 19 test subjects. The normal range (15.4 μmol/L) was indicated in grey.

**Figure 2:**
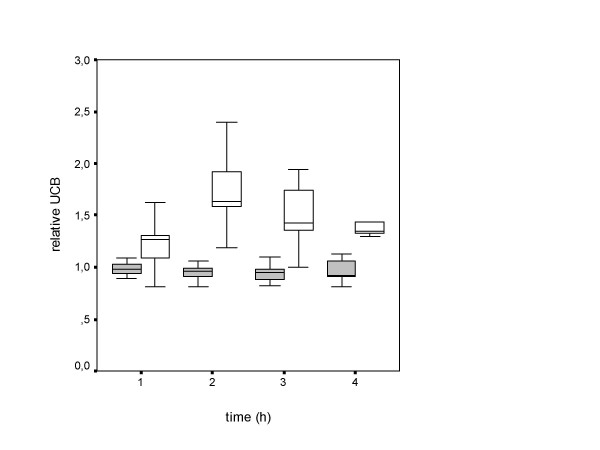
Relative UCB change in nicotinic acid test (solid boxes) and inverse starving test (open boxes) (dotted line indicated the baseline value).

**Figure 3:**
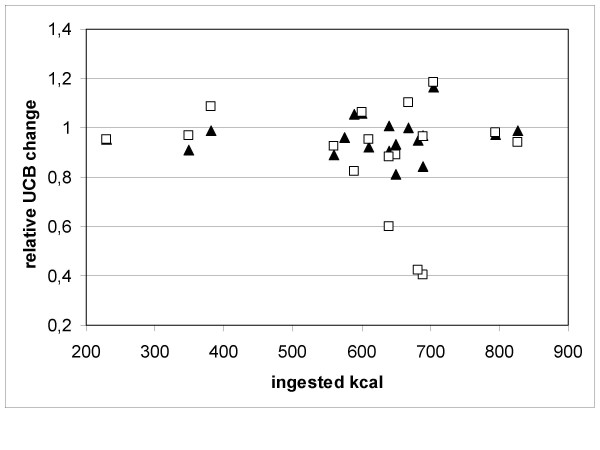
Relative UCB change 2 hours (▲) and 3 hours (□) after lunch. No correlation between ingested kilocalories and the change of the UCB value was found.

The median starving time between last caloric intake at the day before this test and the lunch of choice in the university restaurant was 15:30 hours (range: 13:30–17:15 hours). Side effects were recognized in two patients (table 3). As shown in figure 1, the inverse starving test induced an UCB reduction to normal range (<15.4 μmol/L) or a relative UCB reduction of more than 50% in none of the patients. Box plot analysis revealed only a negligible decrease in UCB levels over the test period (figure 2). We hypothesized that the UCB decline after lunch should be mutually dependent from caloric intake, but no correlation could be detected (Figures 2 and 3).

**Table 3:** Adverse events of the inverse starving test

**Adverse events of the inverse starving test**
1 pt. headache at the evening of the test day
1 pt. vomiting at the afternoon and evening of the test day

### The nicotinic acid test

**Figure 4:**
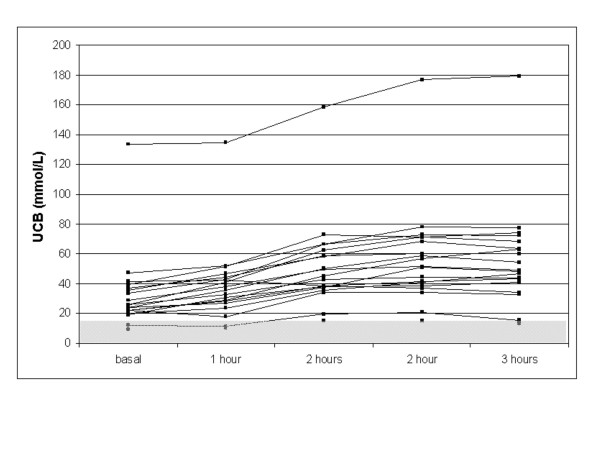
UCB course in the nicotinic acid test in the 19 test subjects. The normal range (15.4 μmol/L) was indicated in grey.

Basal UCB at day 2 (nicotinic acid test) were almost identical (p = 0.88, t-test) with day 1 (inverse starving test). Two hours after ingestion of 170 milligrams NA, UCB was elevated > 50% in 15 of the 18 patients (sensitivity 83%, figure 4). Box plot analysis showed the typical course of nicotinic acid test with a precise discrimination of the relative UCB from the baseline value (figure 2). NA side effects were recognized in 16 patients (table 4). The conjugated bilirubin levels did not change during both tests (data not shown).

**Table 4:** Adverse events of the nicotinic acid test

**Adverse events of the nicotinic acid test**
10 pts. itching skin sensations
9 pts. skin erythema
6 pts. heat waves
5 pts. burning skin sensations
3 pts. Vertigo
2 pts. Nausea
1 pt. impaired nasal ventilation
1 pt. Headache
1 pt. Foot and hand paresthesia

## Discussion

The "inverse starving test" was not able to induce a more than 50% reduction of UCB in our patients with GS. We conclude that this test is not a useful provocation test for GS. As previously reported using intravenous protocols for the nicotinic acid test, the oral ingestion of 170 mg nicotinic acid failed to induce an UCB increase > 50% in 3 of our 18 test persons with GS, which results in a sensitivity of only 83%. Confirming previous findings, this UCB increase was less intensive with the oral than with the intravenous approach, respectively [10,11,18-20].

One potential cause of the failure of our procedure ist, that the inverse starving test was started after only one overnight fast and a number of bilirubin baseline levels were just above normal. In the classic caloric restriction test, h owever, best specificity and sensitivity was achieved after 48 hours [21-23]. It might be speculated therefore, that 48 hours of restricted calorie intake might induce higher baseline bilirubin levels and improve the results of the inverse starving test. However, it was not our aim to introduce another cumbersome and unpleasant procedure to investigate patients for the GS.

Due to a lack of international standards in the evaluation of patients with UCB elevations, patients usually undergo very different investigations to find out the diagnosis of Gilbert's syndrome. In our cohort, patients who consulted their physicians with jaundice were significantly more likely to undergo invasive diagnostic procedures than patients with an incidental finding of elevated UCB. As the median UCB did not differ in these groups, the doctor's decision for an invasive approach seems to be driven by the patient's demand for a diagnosis but not by a substantiated scientific approach.

In conclusion, the inverse starving test is not an appropriate provocation test for patients with suspected GS. Today, no international gold standard exists for the diagnostic criteria of GS and protocols for the nicotinic acid test are differing in a broad range of its test conditions. Due to its unsatisfying sensitivity and its considerable adverse effects, the nicotinic acid test seems not to be mandatory for the GS diagnosis. A growing number of authors suggest that there is no need for provocation tests at all, if no change in the typical laboratory constellation of an isolated UCB elevation occurred after 3–6 months. In agreement with this, we suggest that the clinical diagnosis of GS can be established in patients with a high fraction of UCB, normal values of liver enzymes and no laboratory signs of haemolysis at the time of the initial consultation and three months later.

## Abbreviations

GS: Gilbert's syndrome; UCB: Unconjugated serum bilirubin; ASAT: Aspartate amino transferase; ALAT: Alanine amino transferase; GGT: Gamma-glutamyl transferase; LDH: Lactate dehydrogenase; NA: Nicotinic acid.

## Competing interests

The authors declare that they have no competing interests.

## Authors' contributions

NT and IS conceived of the study and analyzed and interpreted the data. IL and JR conducted the inverse starving test and the nicotinic acid test. MT did the ultrasound examinations. JM has been revised the manuscript critically for important intellectual content. All authors read and approved the final manuscript.
